# IRSEI-based monitoring of ecological quality and analysis of drivers in the Daling River Basin

**DOI:** 10.1038/s41598-024-65511-5

**Published:** 2024-06-24

**Authors:** Jintao Ge, Cheng Qian, Chao Zhang, Li Zhang, Weimin Song, Fuchao Na, Hongwei Ma, Changlai Guo, Shan Jiang

**Affiliations:** 1 Shenyang Center of Geological Survey, China Geological Survey, Shenyang, 110034 China; 2 Institute of Disaster Prevention, Langfang, 065201 China

**Keywords:** IRSEI, The Daling River Basin, Transfer matrix, Geographical detector, Ecological environment, Restoration ecology, Environmental sciences

## Abstract

The Daling River Basin is an important ecological functional area in the western region of Liaoning with outstanding environmental problems. The monitoring of ecological and environmental quality in the basin and the analysis of driving factors are of great importance for the protection of the ecological environment and the improvement of economic quality. In this paper, the three periods of Landsat remote sensing images in 1995, 2010 and 2020 are used as the basic data, and platforms and technical means such as RS and GIS are used to decipher and extract the three periods of land use information, and to construct the land use type transfer matrix. The remote sensing ecological index (RSEI) was improved, and the principal component analysis method was applied to construct the improved remote sensing ecological index (IRSEI) model based on the greenness (NDVI), moisture (WET), heat (LST) and new dryness (N-NDBSI), so as to realize the dynamic monitoring of ecological and environmental quality in the study area. Based on the land use change, combined with the trend of improved remote sensing ecological index (IRSEI) of Daling River Basin, thus achieving the purpose of rapid and efficient dynamic monitoring of ecological quality of Daling River Basin from 1995 to 2020. A geoprobe model was then used to systematically assess the drivers of ecological quality in the catchment. The results show that the improved remote sensing ecological index (IRSEI) can efficiently and accurately obtain the spatial distribution pattern and temporal variation trend of IRSEI in the study area, which is more in line with the characteristics of indicators in this study area. The IRSEI in the study area showed an increasing trend from 1995 to 2020, from 0.4794 to 0.5615, and the proportion of benign ecological classes increased year by year during the period. Among the evaluation indicators, NDVI and N-NDBSI are the main factors affecting the environmental and ecological quality of the Daling River Basin, and the increase of vegetation cover, climate regulation and human activities have obvious promoting effects on the improvement of the ecological environment of the Daling River Basin. This study provides a scientific theoretical basis for the implementation of further ecological environmental protection measures.

## Introduction

The ecological environment is always a necessary condition for human development, the land on which humans depend to survive, and an important guarantee for sustainable economic and social development. Scientific and objective evaluation of ecological environment quality is particularly important, which can provide a strong scientific basis for monitoring, protecting and governing the ecological environment^1–3^. A variety of ecological environment assessment methods have been used to monitor and assess the current state and changes in the quality of the ecological environment, among which the establishment of models such as hierarchical analysis and "pressure-state-response" (PSR) have problems such as subjectivity, difficulty in collecting indicator data, and limited coverage^4,5^. In recent years, with the continuous development of satellite remote sensing technology and the accumulation of massive data, it has been widely used in ecological environment quality analysis and assessment due to its many advantages such as speed, timeliness, objectivity and wide spatial coverage^6^.

So far, remote sensing technology has been one of the important technical means to monitor the ecological environment, in which, for example, long time series observations such as normalised vegetation index (NDVI), net primary productivity (NPP) and so on Refs.^7,8^, these indices can well explain the ecological characteristics of a certain aspect of the ecological environment, but the ecological system has complexity and is affected by a combination of many factors, so it is difficult to make objective evaluations of the ecological environment with a single ecological index^9^. When analysing and researching the ecological environment, it is necessary to conduct a comprehensive analysis of multiple environmental impact factors, rather than studying only a single environmental index and factor, in order to avoid one-sidedness and subjectivity^10^. Remote Sensing Ecological Index (RSEI) is widely used in the evaluation of ecological quality^11^, which integrates four factors, namely vegetation index, surface temperature, humidity and dryness, and is an ecological index that integrates a variety of factors, and is capable of comprehensive evaluation and analysis of ecosystem "health"^10^. Since the RSEI was proposed, it has been widely used in the research of ecological environment quality assessment in various scale areas, such as mining areas and cities^4,12^, due to its advantages of easy data collection, concise process and visualization of results, etc. These researches provide practical references and theoretical support for the ecological environment assessment based on remote sensing.

As the model has been continuously applied to the assessment of ecological environmental quality in different regions, some scholars have adjusted or improved the model based on the characteristics of the study area. The adjustments can be mainly divided into two categories: one is to add and introduce new indicators that fit the characteristics of the study area based on the original indicators, for example, Zhang Wei^13^ included the density of water network as an indicator of ecological environment quality assessment in the context of Hubaoeyu urban agglomerations. Nong Lanping^14^ added humanistic and social development indicators such as population and gross domestic product (GDP). Zhang Jing^15^ introduced aerosol optical thickness (AOD) to construct an improved remote sensing ecological index based on the original model and found that the model can better concentrate the characteristics of the indicators, which can help evaluate the ecological environmental quality of the study area in a more comprehensive way. The second is to choose methods other than principal component analysis to extract remote sensing indexes, Song Meijie^16^ in order to retain richer information under the premise of removing noise interference, its weighted sum of the first three principal components of the principal component analysis output to obtain the final improved remote sensing ecological index. Liu Ying^17^, on the other hand, used kernel principal component analysis (KPCA) instead of PCA to obtain the principal components.

The Daling River is the largest river in the western part of Liaoning Province, and its watershed is part of the fragile ecological environment area in western Liaoning, which is an important ecological functional area in western Liaoning, and the stability of its ecosystem is directly related to the economic development of western Liaoning and the lives of people along the river^18^. The watershed has a complex topography, high mountains and deep valleys, and is affected by anthropogenic and natural factors. Problems such as rock desertification, salinisation and soil erosion are prominent. Therefore, there is an urgent need for research on spatial and temporal changes in environmental quality^19^. In the original RSEI model, the dryness index is represented by the mean value of both the bare soil index (SI) and the building index (IBI). However, the Daling River Basin has a large area and a small proportion of buildings, so using the dryness index constructed by the original method will inevitably lead to data distortion. In addition, the western part of Liaoning has pronounced problems of soil desertification and salinisation and is a semi-arid region. Therefore, the author introduced the soil salinity index instead of the building index and formed a new dryness index with the mean value of both the bare soil index (SI) and the normalised salinity index (NDSI) and constructed the improved remote sensing ecological index (IRSEI). With the help of remote sensing images in 1995, 2010 and 2020, the land use types were obtained by combining manual interactive interpretation, exploring the temporal and spatial changes of land use types, analysing the index changes based on land use changes, exploring the impact of land use on the ecological environment, systematically analysing the process of spatial and temporal variations of ecological environmental conditions and the pattern of ecological environmental conditions under the role of land use, verifying the feasibility of the improved remote sensing ecological indices, providing a new solution for the assessment of environmental quality of the similar semi-arid areas, and providing scientific references for the implementation of further ecological and environmental protection measures in the Daling River Basin^20^.

## Study area and data sources

### Overview of the study area

**Figure 1:**
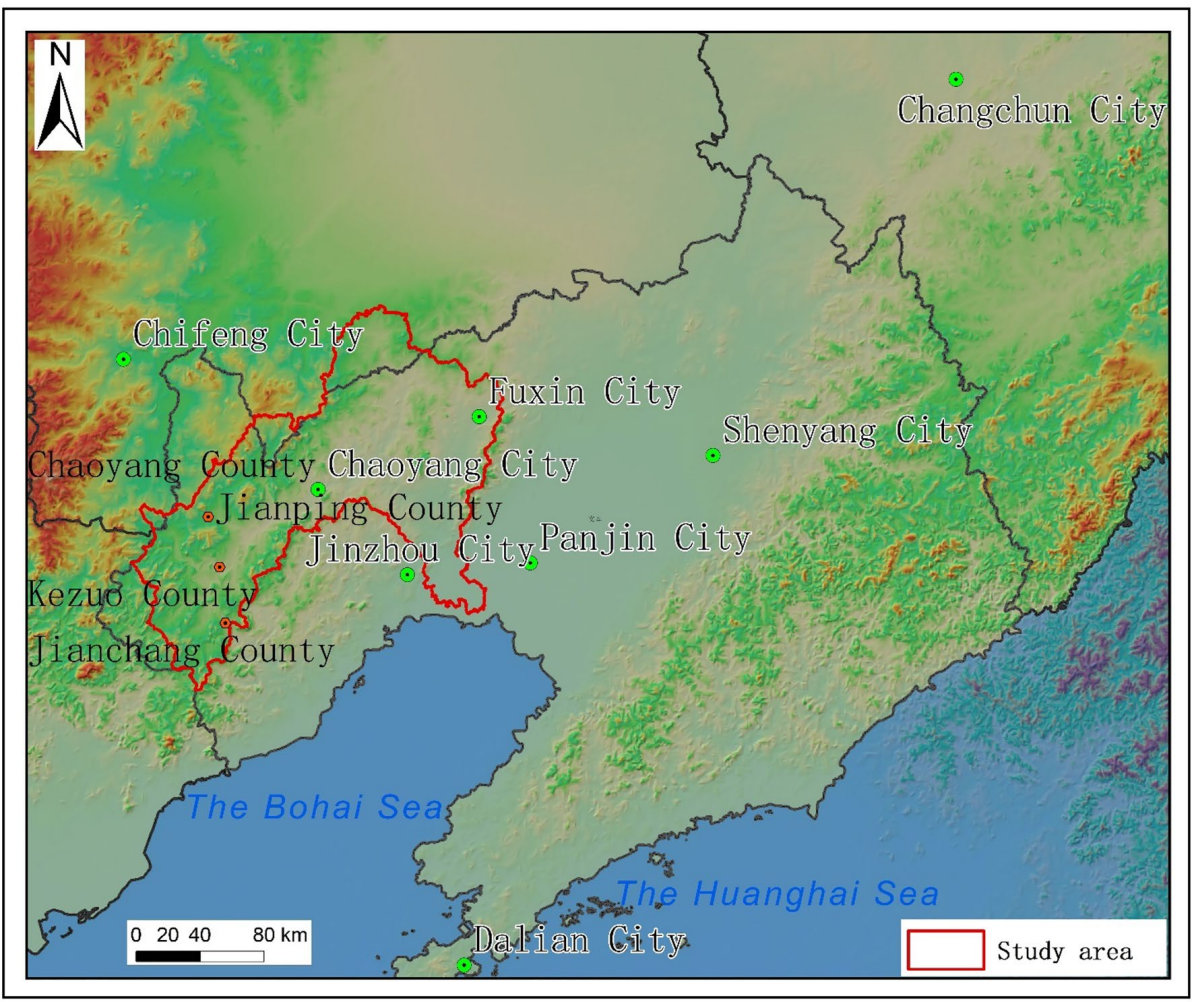
Geographical location map of the study area. Generated in the ArcGIS 10.6 software (www.esri.com).

The Daling River, with a length of 447 km and a watershed area of 23,300 km^2^, is mainly divided into a western and a southern branch, with the western branch originating in Hebei Province and the southern branch originating in Liaoning Province^21^. The geographical extent of the study area is E 119° 06′–121° 54′, N 40° 23′–42° 38′ (Fig. 1). The basin has an average annual runoff of 1.769 billion cubic metres and an average annual precipitation of about 500 mm. It has a typical temperate continental monsoon climate with four distinct seasons, and the overall climate is semi-arid and semi-humid, representative of western Liaoning. The type of landform is mainly hilly and mountainous, with a small number of plains, a high volume of river sand, and severe soil erosion.

### Data sources and pre-processing

The data sources for the remote sensing images are the Landsat 5 TM satellite data in the years 1995 and 2010 and the Landsat 8 OLI satellite images in the year 2020. Landsat images were downloaded from USGS database websites (https://earthexplorer.usgs.gov/). The image quality is good, and the cloudiness of the selected images is less than 5%, which guarantees the accuracy and reliability of the data sources. The imaging period is concentrated in August–September to reduce seasonal differences. Raw data were pre-processed with radiometric calibration, atmospheric correction and geometric correction using the ENVI v. 5.6.2. software (https://www.l3harrisgeospatial.com/Software-Technology/ENVI).

## Research methods

### Land use transfer matrix

The land use transfer matrix is derived from the quantitative description of the system state and state transfer in the system analysis^22^. It reflects information on the transfer of land from each category at the beginning and end of a given period in a given region and is a dynamic process of mutual transformation^23^. The general form of the land use transfer matrix is as follows:

$$Q_{kh}=\left[\begin{array}{c}\begin{array}{cc}{Q}_{11}{Q}_{12} \dots & {Q}_{1n}\\ {Q}_{21}{Q}_{21} \dots & {Q}_{2n}\end{array}\\ \begin{array}{cc}\dots \dots \dots & \dots \\ {Q}_{n1}{Q}_{n2} \dots & {Q}_{nn}\end{array}\end{array}\right]$$

where Q represents area information, n is the number of pre- and post-transfer land use types, k and h (h k, h = 1, 2, …, n) represent pre- and post-transfer land use types, respectively, and Q_kh_ denotes the area of pre-transfer k land use type converted to h land use type^24,25^. According to the LUCC classification standard of the Chinese Academy of Sciences, the land types in the study area were classified into seven first-tier land classes, and the classification system included forest land, grassland, cropland, wetland, watershed, construction land and cropland. The remote sensing interpretation was carried out using human–computer interaction to obtain three phases of land use classification data in 1995, 2010 and 2020. The GIS platform was used to obtain the regional land use transfer matrix from 1995 to 2010 and from 2010 to 2020 by superimposing and statistically analysing the land use information from the three periods.

### Construction of the improved remote sensing ecological index (IRSEI)

According to the improved remote sensing ecological index (IRSEI) proposed in the previous paper, the original remote sensing image data are used, and based on the rapid extraction technology of remote sensing information, the four indices of greenness, heat, humidity and new dryness, which are closely related to human life and activities, are selected, and the indices are obtained with timeliness and high efficiency, which can be a good solution to the basic problem of the difficulty of data collection in monitoring and evaluating the quality of the environment. Specifically, the remote sensing data were used to extract the normalised vegetation index (NDVI) to represent greenness; the remote sensing thermal infrared band data were used to invert the surface temperature (LST) to represent heat; the bandwidth calculation was used to extract the wetness index (WET) to represent moisture; and the bare soil index and the salinity index were calculated and the average of both was taken to obtain the new dryness index (N-NDBSI) to represent dryness. Then, principal component analysis (PCA) was applied to integrate the indicators. PCA (principal component analysis) is an efficient data compression and dimensionality reduction technique that converts the original multi-band image data into a few mutually independent principal components that capture most of the original data characteristics by eliminating redundant information between bands. This method realizes the mapping of high-dimensional data to a low-dimensional space, preserving the main characteristics of the original data points with fewer data dimensions. In constructing a remote sensing ecological index that reflects the quality of the ecological environment, it is crucial to assign appropriate weights to each indicator. The traditional subjective empirical weighting method is convenient but prone to bias. With the PCA method, however, the weights of each indicator can be determined automatically and accurately based on the objective assessment of the contribution of different indicators to the principal components, thus improving the accuracy and reliability of the ecological environment quality assessment. In summary, the standardization of indicators, coupling the four indicator components, through the principal component analysis operation, take the first principal component PCA1 contribution as the weight, to obtain the improved remote sensing ecological index (IRSEI). That is:

$$ {\text{IRSEI }} = {\text{ f }}\left( {{\text{NDVI}},{\text{ LST}},{\text{ WET}}, {\text{N-NDBSI}}} \right). $$

The specific basis for the selection and extraction of the four indicators is as follows:

#### NDVI

Surface vegetation plays the role of "indicator" in the study of global terrestrial ecosystems, and the state of vegetation cover is a visual reflection of the advantages and disadvantages of the ecological environment in a region, and remote sensing technology can directly extract information on the vegetation in a region. NDVI is the most widely used vegetation index, which is closely related to foliage index, plant biomass and vegetation cover. Therefore, NDVI was chosen to represent the greenness index.

#### LST

The thermal indicator, as the name suggests, is the inversion of the surface temperature. Surface temperature is closely related to the growth of vegetation and the circulation of water resources, and is an important indicator for ecological environmental analysis.

#### WET

A humid climate is more suitable for the survival of humans, plants and animals and is one of the conditions for a good ecological environment. The moisture component of coma-cap changes in remote sensing bands (WET) can be a good inversion of soil moisture, and is a better representation of moisture indicators, so WET is used to represent moisture indicators.

#### N-NDBSI

Bare soil is one of the symbols of soil erosion and one of the indicators of the 'dryness' of an area, so the bare soil index (SI) can be a good proxy for the 'dryness' of an area. However, as there are fewer buildings in the study area, the building index (IBI), which is part of the original dryness index (NDBSI) of the RSEI, was removed and replaced by the salinity index (NDSI), which is a good indicator of salinisation in an area. Salinization is one of the serious soil problems in the region, where increased salinity deteriorates the quality of the soil and can even cause desertification of areas. The NDSI can well reflect the "drying" situation of the ground surface and the soil condition in the study area, and can be used as one of the important influencing factors for evaluating the environmental quality of the area. Therefore, the average value of bare soil index (SI) and salinity index (NDSI) was chosen to represent the new dryness index.

The specific formulae for the above indicators are shown in Table 1.

**Table 1:** Calculation methods of indicators.

Indicator	Calculation formula
NDVI	NDVI = (ρ_n_–ρ_r_)/(ρ_n_ + ρ_r_)
LST (TM)	L_6_ = ga·DN + bi T_b_ = K_2_/ln(K_l_/L_6_ + 1) T_s_ = T_b_/[1 + (λT_b_ /α)lnε_6_]
LST (OLI)	L_10_ = τ_10_[ε_10_B_10_(T_s_) + (1–ε_10_) I_10_^↓^] + I_10_^↑^ B_10_(T_s_) = [L_10_–I_10_^↑^–τ(1–ε_10_) I_10_^↓^]/τ_10_ ε_10_ T_s_ = C_1_/{λ_10_ ln{C_2_/{λ_10_^5^[L_10_–I_10_^↑^–τ(1–ε_10_) I_10_^↓^/τ_10_ ε_10_]} + 1}}
WET_TM_ WET_OLI_	WET_TM_ = 0.0315ρ_b_ + 0.2021ρ_g_ + 0.3102ρ_r_ + 0.1594ρ_n_–0.6806ρ_s1_–0.6109ρ_s2_ WET_OLI_ = 0.1511ρ_b_ + 0.1972ρ_g_ + 0.3283ρ_r_ + 0.3407ρ_n_–0.7117ρ_s1_–0.4559ρ_s2_
N-NDBSI	SI = [(ρ_s1_ + ρ_r_)–(ρ_b_ + ρ_n_)]/[(ρ_s1_ + ρ_r_) + (ρ_b_ + ρ_n_)] NDSI = (ρ_r_–ρ_n_)/(ρ_r_ + ρ_n_) N-NDBSI = (SI + NDSI)/2

*ρ*_*r*_, *ρ*_*b*_, *ρ*_*g*_, *ρ*_*n*_, *ρ*_*s1*_ and *ρ*_*s2*_ are the reflectance of TM and OLI in the red, blue, green, near infrared, shortwave infrared1 and shortwave infrared2 bands, respectively. ga and bi are the gain and bias values of the bands, respectively. K_1_ and K_2_ are the calibration parameters. λ is the central wavelength.α is taken as l.438 × 10^–2^ m·K. I_10_^↑^ and I_10_^↓^ are the atmospheric radiant brightness to and from the atmosphere. τ_10_ is the atmospheric transmittance in the thermal infrared band. Ε is Specific emissivity. ε10 is Surface specific emissivity. C_1_ and C_2_ are constants. B10 (Ts) is Brightness of thermal radiation from a blackbody. Ts is the surface temperature.

### The geographical detectors

Geo-detectors provide a mathematical method for revealing the driving forces behind and detecting spatial heterogeneity. The detector is divided into four sections, namely interaction detection, ecological detection, factor detection and risk detection^26,27^. In this paper, we use the factor detection in the geo-detector, and select NDVI, LST, WET and N-NDBSI as indicators, and adopt the degree of explanatory power of IRSEI by single-factor detection, in order to explore the dominant factors affecting the changes of ecological and environmental quality in the Daling River Basin between 1995 and 2020. This is expressed as follows:

$$q=1-\frac{1}{M{\sigma }^{2}}\sum_{k=1}^{R}{M}_{k }{\sigma }_{k}^{2}.$$

Here: the value of q ranges from 0 to 1 and indicates the influence and explanatory power of the indicator on the ecological quality of the watershed, the greater the value of q, the greater the influence of the indicator on the ecological quality of the watershed, the greater the explanatory power. R is the number of indicators selected. M and M_k_ represent the total number of samples and the number of classified samples, respectively. σ^2^ and ${\sigma }_{k }^{2}$ are the variance of the total number of samples and the variance of the classified samples, espectively.

## Results and analyses

### Land use transfer

**Figure 2:**
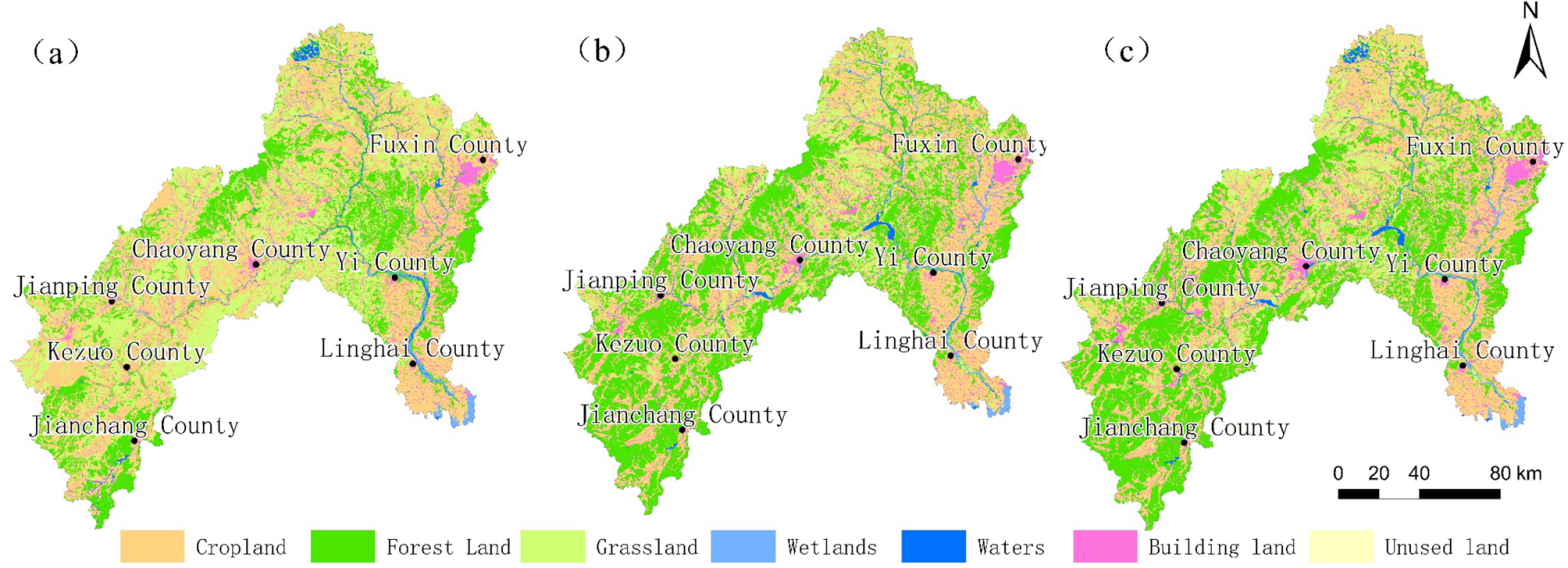
Remote sensing interpretation map of land use types in the Daling River Basin ((**a**) 1995, (**b**) 2010, (**c**) 2020). Generated in the ArcGIS 10.6 software (www.esri.com).

Through the three phases of remote sensing interpretation and comparative study, the land use development pattern was identified, and the land use structure of the Daling River Basin changed in 1995, 2010 and 2020 (Fig. 2). During the period from 1995 to 2010, the area of cropland in the area decreased and the area of forest land and building land increased, of which the area of forest land increased by 4,048.9 km^2^, the area of grassland decreased by 3,555.4 km^2^, and the area of building land increased by 394.3 km^2^. During the period from 2010 to 2020, the land use changes in the area are relatively small of which cropland decreased by 54.3 km^2^, wetlands decreased by 42.43 km^2^, building land increased by 116.2 km^2^ and water area increased by 81.6 km^2^ (see Tables 2 and 3 for details).

**Table 2:** Land use type transfer matrix of the Daling River Basin from 1995 to 2010 (km^2^).

1995 2010	Grassland	Cropland	Building land	Forest land	Wetlands	Waters	Unused land	Total
Grassland	1573.9	1044.6	102.6	3358.8	24.6	10.5	11.8	6126.7
Cropland	586.3	7421.0	533.2	1709.9	143.2	70.6	5.3	10,469.5
Arable land	31.7	225.0	781.6	62.9	10.1	4.0	0.9	1116.3
Forest land	327.4	664.2	83.1	3545.1	41.0	26.1	2.8	4689.7
Wetlands	30.7	98.6	5.8	37.1	235.7	55.6	0.4	463.9
Waters	18.4	70.1	4.0	23.5	71.0	96.3	0.5	283.8
Unused land	2.9	5.1	0.3	1.4	0.1	0.1	6.3	16.2
Total	2571.3	9528.7	1510.6	8738.6	525.7	263.2	28.0	23,166.1

**Table 3:** Land use type transfer matrix of the Daling River Basin from 2010 to 2020 (km^2^).

2010 2020	Grassland	Cropland	Building land	Forest land	Wetlands	Waters	Unused land	Total
Grassland	2332.9	136.1	19.8	62.9	3.4	14.1	2.1	2571.3
Cropland	134.4	9170.5	103.1	54.7	24.2	38.2	3.5	9528.7
Arable land	9.5	35.4	1445.7	16.0	1.6	2.2	0.2	1510.6
Forest land	121.0	91.3	54.7	8455.1	8.7	6.9	0.9	8738.6
Wetlands	7.0	29.7	2.4	7.3	434.8	44.4	0.1	525.7
Waters	1.4	8.5	0.4	1.1	10.7	241.0	0.0	263.2
Unused land	2.2	3.0	0.6	0.5	0.0	0.3	21.4	28.0
Total	2608.6	9474.4	1626.8	8597.6	483.3	347.2	28.2	23,166.1

In order to quantitatively describe the dynamic change characteristics of land use in the Daling River Basin from 1995 to 2020, land use transfer matrices were constructed for the two periods 1995 ~ 2010 and 2010 ~ 2020 (Tables 2 and 3). Based on the transfer matrix analysis^28^, the forest area increased by 4048.9 km^2^ between 1995 and 2010, mainly converted from grassland and arable land. Among them, the area of grassland converted to forest land was 3358.8 km^2^, and the area of cropland converted to forest land was 1709.9 km^2^, mainly in the upper reaches of the Daling River. During the period 2010–2020, 134.4 km^2^ of cropland was converted to grassland, 103.1 km^2^ of arable land was converted to building land, and 54.7 km^2^ of cropland was converted to forest land. At the same time, 62.9 km^2^ of grassland was converted to forest land.

### Construction of the IRESI model

#### Indicator correlation analysis

Principal component analysis (PCA) technique, which aims to utilize the idea of dimensionality reduction to transform multiple indicators into a few composite indicators, has a great advantage in extracting the main information in linearly correlated variables. The data of four indicators, namely greenness, humidity, heat and new dryness, are used to do correlation analysis statistics based on Pearson correlation matrix. As shown in Table 4, the absolute value of the Pearson correlation coefficient of each indicator is also greater than 0.5 at the minimum, and the maximum value is 0.91, and there is a linear or nearly linear relationship between the indicators, which meets the basic requirements for choosing PCA as the integration method.

**Table 4:** Pearson correlation coefficient.

Correlation coefficient	LST	N-NDBSI	WET	NDVI
LST	1.00			
N-NDBSI	0.58	1.00		
WET	− 0.56	− 0.91	1.00	
LST	− 0.54	− 0.89	0.72	1.00

#### Principal component analysis before and after RSEI model improvement

RSEI (based on four indices of greenness, humidity, heat and dryness) and IRSEI (based on four indices of greenness, humidity, heat and new dryness) were constructed for the study area respectively, and the principal component analysis characteristics of the two models were compared (Table 5).

**Table 5:** Comparison of principal component analysis characteristics between RSEI and IRSEI.

PCA results	Models	Year		
		1995	2010	2020
Eigenvalue	RSEI	0.016	0.013	0.021
	IRSEI	0.027	0.023	0.037
Contribution rate/%	RSEI	56.34	77.95	78.64
	IRSEI	68.47	84.57	86.42

As for the contribution of the first principal component PC1: the contribution of the first principal component of the RSEI model ranged from 56.34 to 78.64%, with an average contribution of 70.98%, and the difference between the maximum and minimum values was 22.30%. The contribution rate of the first principal component of the IRSEI model ranges from 68.47 to 86.42%, with an average contribution rate of 79.82%, which is 8.84% higher than that of the RSEI model, and the difference between the maximum and minimum values is 17.95%, which is smaller than the difference of the RSEI model, although there are fewer data, it can be shown that the contribution rate of the first principal component of the IRSEI model is more stable than that of the RSEI model. In addition, a comparison of the PC1 contribution rates in the same years shows that the IRSEI model has a higher contribution rate than the RSEI model in all three pairs of data. This further indicates that the IRSEI model is better than the RSEI model in terms of the contribution of the first principal component.

As for the first principal component eigenvalues: The first principal component eigenvalues of the RSEI model range from 0.013 to 0.021, and the first principal component eigenvalues of the IRSEI model range from 0.023 to 0.037.A comparison of the first principal component eigenvalues of the two models in each year reveals that the first principal component eigenvalues of the IRSEI model are higher than the first principal component eigenvalues of the RSEI model. The magnitude of the eigenvalues indicates the amount of information contained in the eigenvectors, and by comparing the eigenvalues of the two models, it can be concluded that the IRSEI model better synthesizes the characteristics of each indicator.

#### Results of the IRSEI evaluation

**Figure 3:**
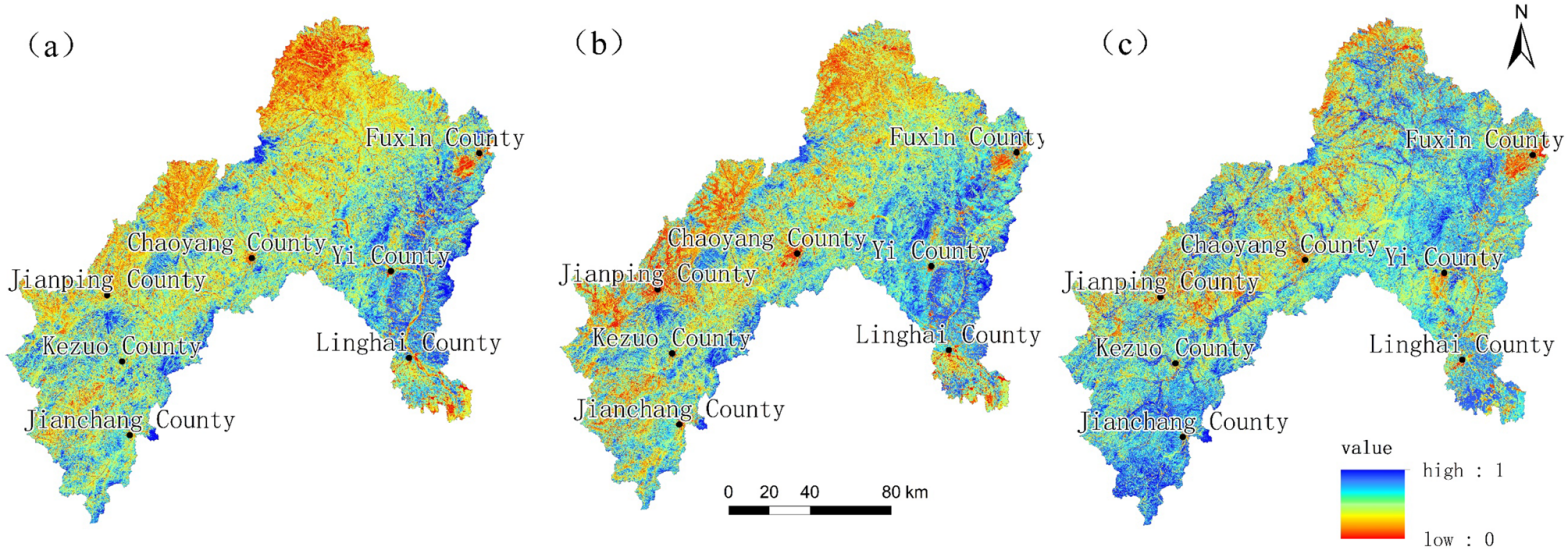
Improved remote sensing ecological index (IRSEI) evaluation maps of the Daling River Basin ((**a**) 1995, (**b**) 2010, (**c**) 2020). Generated in the ArcGIS 10.6 software (www.esri.com).

Using ENVI v. 5.6.2. software (https://www.l3harrisgeospatial.com/Software-Technology/ENVI) as the remote sensing data processing platform and ArcGIS software as the data analysis and thematic mapping tool, the improved remote sensing ecological index was extracted based on the three periods of remote sensing images in 1995, 2010 and 2020 by coupling the four indicators of greenness, heat, humidity and new dryness, which are shown in Fig. 3 as well as Table 6.

**Table 6:** Statistical table of evaluation indicators for remote sensing index in the Daling River Basin.

Year	Indicator				
	Greenness	Heat	Humidity	New dryness	IRSEI
1995	0.6175	0.3721	0.6374	0.5671	0.4794
2010	0.6456	0.4655	0.6216	0.5831	0.4972
2020	0.7154	0.3895	0.6019	0.5763	0.5615

As can be seen from Table 6, the IRSEI in the study area showed an increasing trend from 1995 to 2020, with an increase of 17.13%, during which the proportion of benign ecological classes increased year by year, and the remotely sensed ecological index increased slightly from 1995 to 2010, and then increased significantly in the area from 2010 to 2020. The upward trend of the greenness value (NDVI) in the Daling River Basin from 1995 to 2020 can be attributed to the substantial increase in the area of forest land, which indicates that the effect of afforestation has been shown, especially in the north-western part of the study area, where the NDVI has increased significantly in comparison with other regions, which is in line with the above law of land use development, and a steady increase in the area of forest land can be seen. From 1995 to 2020, the humidity (WET) indicator in the Daling River Basin shows a decreasing trend, and the overall drought situation in the basin is becoming more serious year by year, with evaporation being greater than precipitation, and the water storage capacity is weak. In the case of decreasing humidity, dryness should increase, but the new dryness (N-NDBSI) from 2010 to 2020 is also decreasing, indicating that the salinity index (NDSI) in its indicator is significantly decreasing, which indicates that land salinisation in the study area has been significantly controlled during this period.

### Spatio-temporal characterisation of IRSEI

The ecological environment of the Daling River Basin was classified into five grades of poor (IRSEI < 0.2),bad (0.2 ≤ IRSEI < 0.4), fair (0.4 ≤ IRSEI < 0.6), good (0.6 ≤ IRSEI < 0.8) and excellent (IRSEI ≥ 0.8) at 0.2 intervals^29^.The area and proportion of each grade of IRSEI are shown in Table 7.

**Table 7:** Area and proportion of each level of IRSEI.

IRSEI ratings	1995		2010		2020	
	Area/km^2^	Proportions/(%)	Area/km^2^	Proportions/(%)	Area /km^2^	Proportions/(%)
Poor [0 ~ 0.2)	2198.53	9.49	3074.48	13.27	2077.48	8.97
Bad [0.2 ~ 0.4)	6418.00	27.70	5001.08	21.59	3413.18	14.73
Fair [0.4 ~ 0.6)	7621.36	32.89	6614.42	28.55	5866.41	25.32
Good [0.6 ~ 0.8)	5165.98	22.30	5568.77	24.04	6881.25	29.70
Excellent [0.8 ~ 1.0]	1764.98	7.62	2910.17	12.56	4930.63	21.28

**Figure 4:**
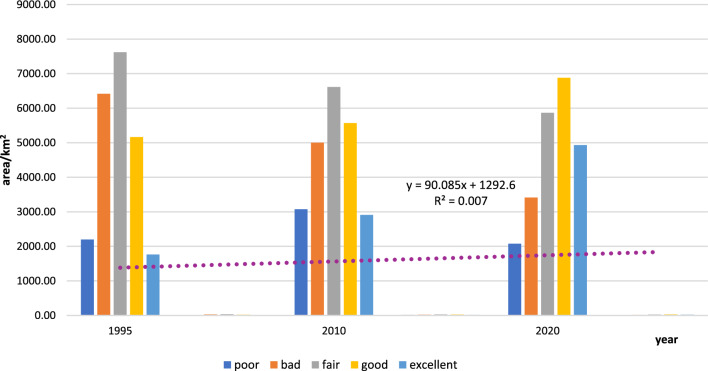
Ecological environment area at different levels in the Daling River Basin. Generated in the Microsoft Office Word 2023 (https://www.microsoft.com).

From 1995 to 2010, the increase in IRSEI was 3.71%, which was not obvious. From Table 7 and Fig. 4, compared to 1995, the proportion of the area with poor quality increased from 9.49 to 13.79% in 2010, while the proportion of the area with excellent quality also increased from 7.62 to 12.56%, i.e. the ecological environmental quality is two-level differentiation, although stable. From 2010 to 2020, the IRSEI increased significantly by 12.93%, the area of the area with excellent grade increased significantly from the proportion of 12.56% to 21.28%, and the proportion of the area with poor grade also decreased from 13.27 to 8.97%, compared to 1995 to 2010, 2010 to 2020 is the overall environmental quality is improving.

Based on the overall analysis of land use transfer matrix and IRSEI spatio-temporal characteristics, the author believes that the reason for the above situation is that the Daling River Basin land use type is dominated by forest land and cultivated land, and as a whole, the area of cultivated land decreased during the period 1995–2020, and the area of forest land and grassland increased significantly, and the conversion of land use type was obvious. From the point of view of the specific transfer of land use, during the period from 1995 to 2010, the conversion between land use types has been more frequent, and the ecological management in this phase has achieved certain results, while the social economy has also developed rapidly, but there are some problems in this process, such as the lack of rationalisation of land use, there is a waste of resources, environmental damage and other phenomena. Since 2010, the transition between land categories has gradually become stable. During this period, the blind development of arable land, as well as land degradation, soil erosion and salinisation, have been effectively managed and controlled, and as a result, the quality and utilisation rate of land have been significantly improved, and these improvements have led to an overall improvement in the quality of the ecological environment.

Throughout the study period, all cities and counties showed some increase in IRESI, indicating a significant improvement in the quality of the ecological environment within the county. Especially in Jianchang County, Kazuo County and Linghai City, the ecological environment quality has improved significantly, which fully proves the effectiveness of the ecological management project in improving the ecological environment quality. From a spatial perspective, the magnitude of IRESI changes in cities and counties gradually increased from southeast to northwest, and the ecological environment conditions of cities and counties in the southern coastal and southeastern regions were significantly better than those in the central part of the watershed and the hilly areas in the north. Therefore, we should pay special attention to the central and northern regions of the watershed in the future management of soil erosion and land salinisation.

### IRSEI response to drivers

**Figure 5:**
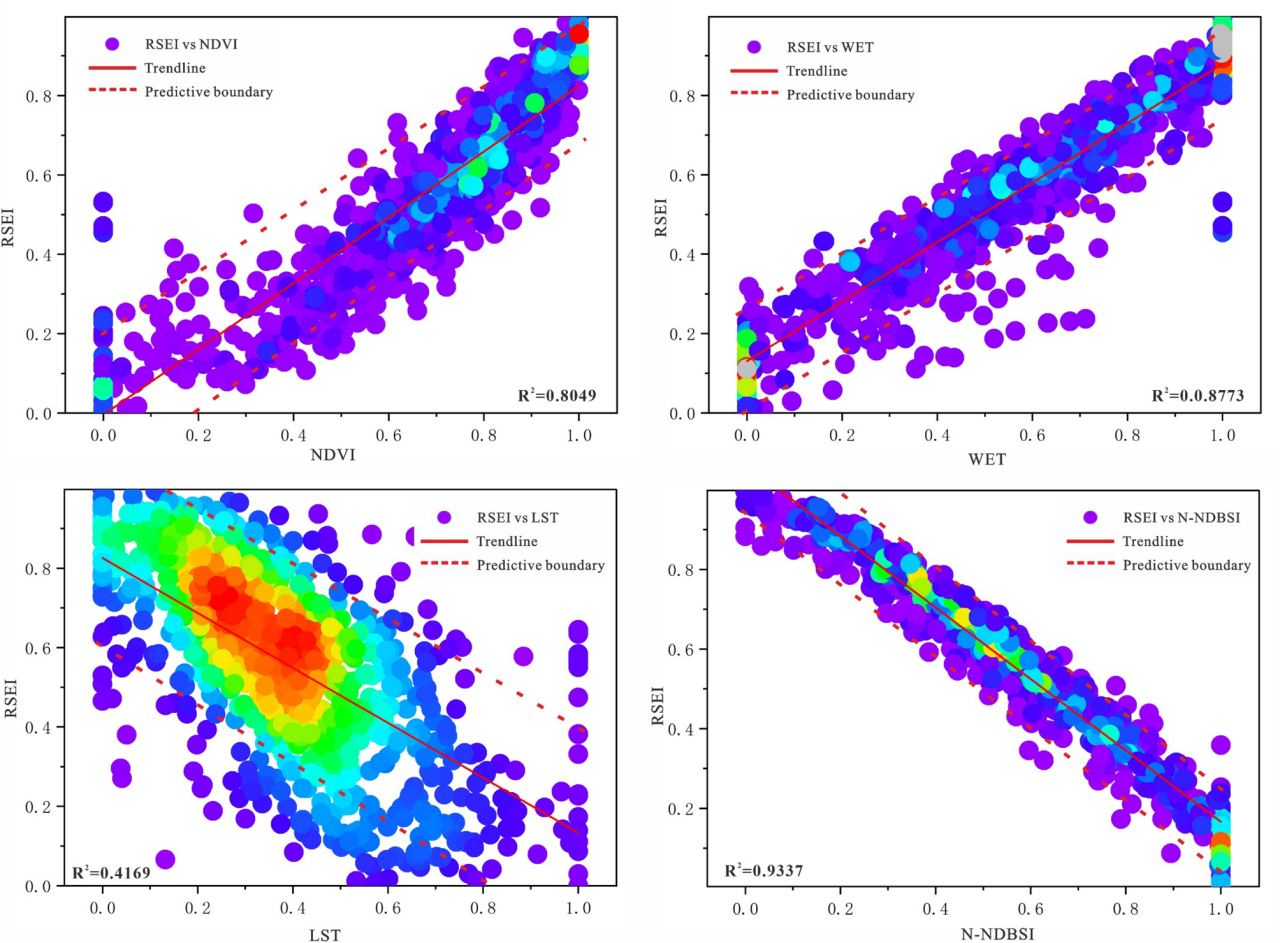
Scatter density maps of NDVI, WET, N-NDBSI and LST and IRSEI at the sampling points. Generated in the Origin 2023 (https://www.originlab.com).

Spatial correlation analyses of ecological and environmental quality in the Daling River Basin were analysed for quantitative research. Sampling was carried out in the whole study area based on a 5 km × 5 km grid, with 972 sampling points arranged in each layer. Based on all sample point data in multiple years, the corresponding scatter density plots were plotted to determine the correlation between the drivers and the IRSEI^30^. As shown in Fig. 5, with the exception of a few anomalies, the sample points were almost all within 90% of the predicted limits, and the scatter plot was accurate. The IRSEI increased with increasing values of NDVI and WET, and decreased with increasing values of LST and N-NDBSI, indicating the positive correlation of NDVI and WET with the environmental quality of the Daling River Basin, and the negative correlation of LST and N-NDBSI with the environmental quality of the Daling River Basin.

The geo-detectors were introduced to reveal the factors affecting the quality of the ecological environment, with NDVI, LST, WET and N-NDBSI as the independent variables (factors) and IRSEI as the dependent variable. The results of the single-factor detections are shown in Table 8, and the p-values of the indicators are less than 0.01, which indicates that the detections are statistically significant, and the q-values are in line with the significance test. The 3-year q-means of NDVI, WET, LST and N-NDBSI were 0.864, 0.623, 0.631 and 0.766, respectively, with an explanatory power of more than 50.0% on the ecological environment of the Daling River Basin, indicating that the four indicators play an important role in influencing the ecological quality. Among them, the NDVI indicator with the largest q-mean and an explanatory power of more than 85.0% is the dominant factor influencing the ecological quality of the environment in the Daling River Basin, indicating that greenness has the greatest influence on the ecological quality of this basin. Surface vegetation can play a role in soil fixation and reduce soil erosion, slow surface runoff and regulate surface moisture; and protect ecological diversity and maintain ecosystem balance, thereby reducing the incidence of environmental problems. The mean q-value of the N-NDBSI indicator is 0.766, with an explanatory power of more than 75.0%, which is a secondary driving factor, indicating that promoting the management of soil salinisation, desertification and surface drying and building a new type of city are also necessary to improve the quality of the ecological environment. The explanatory power of the effects of WET and LST on the ecological environment of Daling River Basin is 62.3% and 63.1%, respectively, from which it can be inferred that the changes in environmental humidity and heat affect the vegetation cover and thus the quality of the ecological environment.

**Table 8:** Results of detecting the impact factors of ecological environment quality.

Impact factor	1995		2010		2020		q average
	q	p	q	p	q	p	
NDVI	0.865	0.000	0.849	0.000	0.878	0.000	0.864
WET	0.603	0.000	0.626	0.000	0.639	0.000	0.623
LST	0.647	0.000	0.602	0.000	0.645	0.000	0.631
NDBSI	0.753	0.000	0.789	0.000	0.757	0.000	0.766

## Conclusion

Improved remote sensing ecological index (IRSEI), which can efficiently and accurately obtain the spatial distribution pattern and temporal variation trend of IRSEI in the study area, and compared with the original RSEI model, the IRSEI model better synthesizes the characteristics of the indicators, which is more in line with the characteristics of the study area, and it can provide a new scheme for the assessment of the environmental quality of the similar semi-arid areas.

The IRSEI of the study area from 1995 to 2020 showed an upward trend, from 0.4794 to 0.5615, with an increase of 17.13%. Land space and land use have been planned more efficiently and rationally, effectively controlling the occurrence of land degradation and soil erosion, and contributing to the overall improvement of ecological environmental quality.

The NDVI is the dominant factor influencing the environmental and ecological quality of the Daling River Basin, and the N-NDBSI indicator is the secondary driver. In terms of influencing factors, the increase in vegetation cover, climate regulation and human activities have a significant effect on the improvement of the ecological environment in the Daling River Basin.

The research in this paper has the limitation of less validation data, the assessment of environmental quality of a region needs more comprehensive support of big data, continue to improve the improved remote sensing ecological index (IRSEI) model and explore the validation method of the model is the author's next focus on the direction of research.

## Data Availability

The datasets used and/or analysed during the current study available from the corresponding author on reasonable request.
